# Differential expression of breast cancer-associated genes between stage- and age-matched tumor specimens from African- and Caucasian-American Women diagnosed with breast cancer

**DOI:** 10.1186/1756-0500-5-248

**Published:** 2012-05-22

**Authors:** Jessica M Grunda, Adam D Steg, Qinghua He, Mark R Steciuk, Suzanne Byan-Parker, Martin R Johnson, William E Grizzle

**Affiliations:** 1 Department of Medicine, Division of Endocrinology, University of Alabama at Birmingham, Birmingham, AL, 35294, USA; 2 Department of Obstetrics and Gynecology, University of Alabama at Birmingham, Birmingham, AL, 35294, USA; 3 Department of Chemical Engineering, Tuskegee University, Tuskegee, AL, 36088, USA; 4 Department of Pathology, University of Alabama at Birmingham, Zeigler Research Building, 703 South 19th Street, ZRB 408, Birmingham, AL, 35294, USA; 5 Department of Pharmacology and Toxicology, University of Alabama at Birmingham, Birmingham, AL, 35294, USA

**Keywords:** Breast cancer, Gene expression, Race, Estrogen signaling, Cell cycle, Cell migration

## Abstract

**Background:**

Recent studies suggest that the poorer breast cancer outcome observed in African-American women (AAW) may, in part, result from underlying molecular factors. The purpose of this study was to investigate gene expression differences between Caucasian-American women (CAW) and AAW that may contribute to this poorer prognosis.

**Methods:**

The expression of 84 genes involved in breast carcinoma prognosis, response to therapy, estrogen signaling, and tumor aggressiveness was assessed in age- and stage-matched CAW and AAW paraffin-embedded breast cancer specimens. The Wilcoxon–Mann–Whitney Test was used to identify genes with a significant difference in expression between CAW and AAW. To determine if the differentially expressed genes could segregate between the CAW and AAW, we performed semi-supervised principal component analysis (SSPCA).

**Results:**

Twenty genes were differentially expressed between AAW and CAW. SSPCA incorporating these 20 genes segregated AAW and CAW into two distinct groups. AAW were significantly (p < 0.05) more likely to display aberrations in G_1_/S cell-cycle regulatory genes, decreased expression of cell-adhesion genes, and low to no expression of ESR1, PGR, ERBB2 and estrogen pathway targets.

**Conclusions:**

The gene expression differences identified between AAW and CAW may contribute to more aggressive disease, resistance to therapy, enhanced metastatic potential and poor clinical outcome. These findings support the hypothesis that breast cancer specimens collected from AAW display distinct gene expression differences compared to similar tissues obtained from CAW. Additional population-based studies are necessary to determine if these gene expression variations contribute to the highly aggressive and treatment-resistant breast cancer phenotype frequently observed in AAW.

## Background

Multiple studies have found distinct ethnic disparities in breast cancer outcome between African-American women (AAW) and Caucasian-American women (CAW) in incidence rate, age-of-onset, mortality and survival. Although the overall incidence rate for breast cancer is higher in CAW compared to AAW, the age-adjusted mortality rate for AAW (33/100,000) is significantly higher than any other ethnic group examined, including women of Caucasian descent [1]. In fact, AAW at all breast cancer stages assessed (localized, regional & distant) have a much lower 5-year survival rate (78%) compared to CAW (90%) [2]. Although breast cancer risk increases with age in all ethnicities, women of African-American ancestry are more often diagnosed at a much younger age, with 30–40% of AAW diagnosed with breast cancer prior to 50 years of age, compared to just 20% for CAW [3]. This trend is even more striking considering AAW are diagnosed more frequently with higher-grade tumors that are resistant to traditional therapies [4]. The mechanisms underlying poorer outcome in AAW diagnosed with breast cancer remains to be elucidated.

Early studies suggested that the poorer outcome observed for AAW diagnosed with breast cancer resulted from disparities in social economic status (SES), education level, access to health care, diet, religious beliefs, and geographical location [5-9]. However, recent research suggests that differences in clinical outcomes likely arise from both societal and genetic factors. Several large population-based meta-analyses report that AAW display a significantly higher mortality rate than when compared to any other ethnicity, even after accounting for SES [10,11]. Additionally, even when African and Caucasian-American women had equal access to health care and/or underwent identical treatment regimens, the disparity in patient outcome persisted. For instance, two Department of Defense studies examining treatment outcome in breast cancer patients found that mortality rates were still significantly higher in AAW versus CAW even though patients had equal access to health care and underwent identical treatment regimens [12,13]. Another study by Albain et al. investigating survival of breast cancer patients enrolled in randomized clinical trials of the Southwest Oncology Group found that overall survival rates for African American patients were significantly poorer, even though patients received the same treatment regimens and were controlled for both prognostic factors and SES [14]. These studies collectively support the hypothesis that while there are sociological factors contributing to the higher mortality rates seen in AAW, other causative factors exist.

A mounting body of evidence now suggests that women of African-American ancestry may harbor a greater genetic predisposition for a more aggressive breast cancer phenotype. Recent studies have demonstrated that young, premenopausal women of African American descent are more likely to display histological characteristics depictive of the basal-like subtype of breast cancer, known for its aggressive behavior and poorer clinical outcome, compared to any other age group of any other ethnic background examined [15,16]. Histologically, breast carcinomas from AAW more often display pushing, non-infiltrative tumor margins, nuclear pleomorphism, lymphocytic infiltrate, large primary tumors, necrosis, lack of tubule formation, as well as high mitotic indices and histological grade [15,17-21]. In addition, AAW are more likely to lack expression of both the estrogen and progesterone receptors, and often display the triple negative phenotype (ER-, PR-, HER2-), thus precluding treatment with such targeted therapies as tamoxifen, anastrozole, and Herceptin [17,20,22]. Women of African-American ethinicity are also more likely to exhibit specific alterations in the levels of genes involved in cell-cycle regulation and apoptosis, including higher quantities of p16, p53, and cyclin E, and lower levels of BCL-2, cyclin D1, and p27 [20,22]. In addition to these ‘basal-like’ features, other studies have found that AAW had a significantly higher prevalence of deleterious mutations in *BRCA1* and *BRCA2* compared with CAW [23].

Many of these genetic differences may be associated with differences in tumor grade and hormone receptor status (ER and HER-2/neu), both of which have been found to independently influence gene expression profiles. Although, a study recently published by Field et al. determined that even when patient tissues were matched on age, grade, and estrogen receptor status, significant differences in gene expression profiles were still observed [24]. The genes identified spanned a diverse array of cellular functions including the proteasome system, eye lens physiology, cell growth and differentiation, and cellular immunity and inflammation [24]. While these studies collectively provided insight into potential biological factors contributing to the poor clinical outcome of AAW, further studies are needed to clarify the role of genetics in AAW breast cancer epidemiology.

The identification of potential gene expression differences driving the disparities in health outcome between AAW and CAW is critical to improving the treatment response and survival of these women as these molecular differences may impact breast cancer prevention, screening practices, diagnostic testing and treatment protocols. In this study we utilized a novel experimental approach to investigate differences in gene expression between AAW and CAW, independent from age or disease stage at diagnosis. Paraffin-embedded, age- and stage-matched breast carcinoma samples from AAW and CAW patients were macrodissected to enrich the specimens in tumor cell content (>80%). Gene expression analysis of genes previously implicated in breast cancer prognosis, treatment response, estrogen signaling, and tumor aggressiveness was performed using The Human Breast Cancer and Estrogen Receptor Signaling RT^2^ Profiler PCR Array from SABiosciences. Using the Wilcoxon-Mann–Whitney Test we identified genes displaying a significant difference in expression between tumors obtained from AAW and CAW breast cancer patients. These analyses identified a distinct molecular profile in women of African-American descent, often associated with the basal-like phenotype and previously associated with resistance to therapy and poor clinical outcome, supporting the hypothesis that AAW may have a gene-expression based predisposition for a more aggressive disease phenotype.

## Methods

### Selection of cases

Consent was obtained for all patients prior to start of study. The use of human tissues was approved by and conducted in accordance with the policies of the Institutional Review Board at the University of Alabama at Birmingham. The records of archival cases of breast cancer from UAB Surgical Pathology were searched to identify age (± 5 years) and stage (assessed by a pathologist) matched cases of ductal carcinoma. The quality of the available archival blocks from the matched cases was then assessed through hematoxylin and eosin (H + E) staining of newly cut sections from the original diagnostic blocks. Sections were examined and selected for areas of tumor that could be macrodissected into greater than 80% invasive cancer.

### Analysis of receptor status

ER, PR and HER2 status were determined in the CAP/CLIA accredited laboratory of University of Alabama at Birmingham Hospital with the exception of one case. Immunostaining of ER (Clone SP1) and PR (1 E2) was performed using a semi-automated immunostainer (Ventana, Model XT) and an Ultraview HPR Multitimer approach. Tumors were considered positive if 1% or greater of tumor cells stained. Percentage of staining as well as intensity 1+ (weak) to 3+ (strong) was also reported. The HER2/neu status was determined by CISH using the Spot-Light Kit (Invitrogen), which is specific for the HER2 gene locus on chromosome 17q11.2-21. A minimum of 30 tumor cell nuclei were evaluated per patient. Criteria are as follows: ≥ 6 dots in the majority of carcinoma cells is amplified, 4–6 is equivocal, and ≤ 4 dots is non-amplified. All evaluations are done by standard microscopy. Cases prior to 2005 were evaluated by immunohistochemistry where 0 was negative if no staining or membrane staining in less than 10% of tumor cells, 1+ was negative if weak membrane staining in greater that 10% of tumor cells, 2+ was positive if weak to moderate complete membrane staining in greater than 10% of tumor cells, and 3+ was positive if strong complete membrane staining in greater than 10% of tumor cells [25,26].

For the one case in which ER, PR, HER2/neu clinical data was unavailable, immunostaining was performed in Dr. William Grizzle’s research laboratory using ER alpha (clone SP1), PR alpha and beta (clone PgR 636), and HER2/neu (clone 3B5) and evaluated by a pathologist.

### Sample preparation

#### Macrodissection

An H + E section was matched and orientated to the paraffin block from which it was cut and areas of benign tissue and non-invasive neoplasms were identified and removed so that after macrodissection, the ductal carcinoma remaining in the block contained at least 80% ductal carcinoma. The tumor areas were re-embedded and new H + E sections were cut to confirm that the ductal carcinoma was successfully enriched by the macrodissection. Ten 10-μm sections were then cut for RNA extraction.

#### RNA extraction

Paraffin tissue curls were deparaffinized as previously described [27]. Total RNA isolation was then performed using the Roche High Pure RNA Paraffin Kit (Roche Diagnostics, Manheim, Germany) as per manufacturer’s instructions. Total RNA was eluted in 30 μl of RNase-free water and stored at −80°C until further analysis. The concentration of all RNA samples was quantitated through linear regression analysis of a standard curve derived from known concentrations of normal breast RNA. *Ribosomal protein, large, P0* (*RPLP0*), which has been previously validated by our laboratory [28], was used as the housekeeping gene.

#### Reverse transcription

Complementary DNA was prepared using the RT^2^ First Strand Kit (SABiosciences, Frederick, MD) as per manufacturer’s instructions. Approximately 0.5 μg of total RNA from each sample was used for cDNA synthesis.

### Analysis of samples by the RT^2^ profiler PCR array

The pre-designed Human Breast Cancer and Estrogen Receptor Signaling RT^2^ Profiler PCR Array (SABiosciences) was utilized to simultaneously analyze 84 genes related to breast cancer regulation and estrogen receptor-dependent signal transduction in cDNA samples. The housekeeping genes *B2MHPRT1RPL13AGAPDH* and *ACTB* are included on each Array. Each cDNA sample was added to 2X SuperArray RT^2^ qPCR Master Mix (SABiosciences) and 25 μl of the mixture was added to each well of the PCR Array using an eight-channel pipettor. The plate was sealed and PCR amplification was performed using an Applied Biosystems Prism 7900HT sequence detection system. Thermal cycler conditions were as follows: 2 minutes at 50°C, 10 minutes at 94.5°C, then 40 cycles of 30 seconds at 97°C and 1 minute at 59.7°C. Delta cycle threshold (Delta C_T_) and expression values were calculated using the comparative cycle threshold (C_T_) method as previously described by our laboratory [27,29].

### Statistical analysis

#### Fisher’s exact test

Fisher’s exact test is a statistical significance test used in the analysis of contingency tables to calculate whether there is a significant association between categorical variables. It is employed when sample sizes are small so the normal approximation and chi-square calculations are not accurate [30].

#### Wilcoxon–Mann–Whitney test

In order to determine what genes were differentially expressed between the CAW and AAW, we utilized the Wilcoxon–Mann–Whitney test [31] using gene ΔC_T_ values. The Wilcoxon–Mann–Whitney test examines the null hypothesis that gene expression levels in the two groups (CAW and AAW) are independent samples from identical continuous distributions with equal medians, compared against the alternative that they do not have equal medians. Each gene is evaluated independently to determine the statistical significance of the difference between the two-group medians. A p-value of < 0.05 was considered statistically significance in this study.

#### Semi-Supervised Principal Component Analysis (SSPCA)

To determine if the genes identified through the Wilcoxon–Mann–Whitney test could visually segregate the AAW and CAW into two distinct groups we performed SSPCA [32,33]. In traditional PCA, all gene expression values are used to identify combinations of genes that separate samples into distinct groups. SSPCA has the advantage of using only those genes previously associated with clinical and/or demographic factors, and, in this case, patient ethnicity, to segregate samples into subgroups, thus allowing clear visualization of how gene expression patterns segregate groups without interfering background noise from genes that are not differentially expressed. The Komogorov-Smirnov normality test was applied to the identified principle components (PCs) to ensure the data was approximately normally distributed [34].

#### Pearson correlation

To calculate the strength and direction of the linear association between the expression of gene pairs across all samples, and/or the AAW and CAW patient subpopulations individually, we used Pearson’s Correlation. Pearson’s correlation assumes a Gaussian distribution of gene expression values within sample sets (i.e. AAW patients).

#### Hotelling’s T^2^ test

Hotelling’s T^2^ test is used in multivariate hypothesis testing, which is a generalization of Student’s *t* test in univariate hypothesis testing [35]. Given the case of p-variate observations from two multivariate normally-distributed populations with common covariance matrix, Hotelling’s T^2^ statistic can be used to test the equality of the vector of means associated with the two samples [35]. In this work, we apply Hotelling’s T^2^ test to examine the multivariate gene differences between AAW and CAW patients.

## Results

### Patient characteristics

Both age and disease stage at diagnosis are potential factors influencing the poorer outcome observed in women of African-American descent. To ensure that any gene expression differences identified in this study were not due to age or disease stage, we selected age- and stage-matched paraffin-embedded samples of ductal breast carcinoma samples from African- and Caucasian-American women using archival records stored at the University of Alabama at Birmingham. From 80 matched archival specimens surveyed, 12 pairs were deemed of high enough quality for macrodissection and future study. All samples examined in this study were collected through biopsy or tumor resection prior to start of radiation, chemotherapy, or other therapies. Patient treatment was not significantly different between AAW and CAW for the 24 patients, and consisted mainly of lumpectomy, total mastectomy, radiotherapy, tamoxifen, and arimidex. Due to the short amount of elapsed time between initial patient diagnosis and study analysis, associations between clinical parameters (race, age, disease stage, and treatment) and patient survival could not be determined.

As illustrated in Table 1, AAW were more likely to be negative for ER, PR, and HER2, however this observation was not significant, perhaps owing to small number of samples examined in this study. In contrast, patient ethnicity was significantly associated with tumor grade (p = 0.0131) with AAW patients more often displaying a higher tumor Grade (Table 1).

**Table 1:** Unmatched characteristics in the study population

**Characteristic**	**AAW (n = 11)**		**CAW (n = 11)**	
Receptor Status				
ER	6	55%	10	91%
PR	5	45%	7	64%
HER2	5	45%	7	64%
Tumor grade				
Well	0	0%	2	18%
Moderate	2	18%	7	64%
Poor	9	82%	2	18%
Bloom and Richardson Score				
I	0	0%	2	18%
II	2	18%	7	64%
III	9	82%	2	18%

### Gene expression differences between AAW and CAW

**Figure 1:**
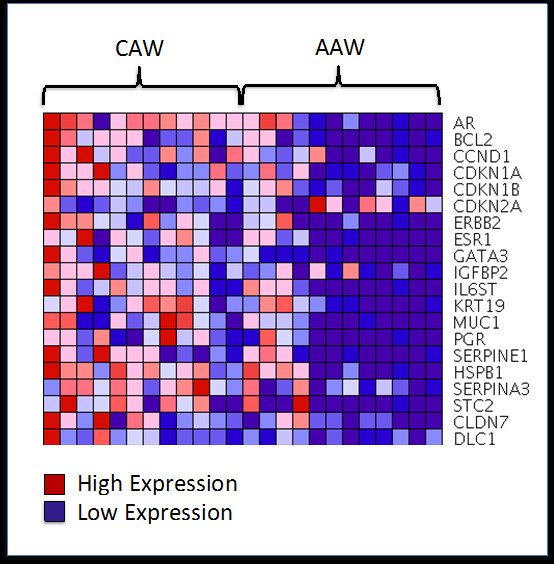
Heat map illustrating differences in the expression of 20 breast cancer-associated genes between AAW and CAW breast cancer patients.

Previous studies suggested that AAW women might have a genetic predisposition for a more aggressive breast cancer phenotype. To investigate this possibility further, we examined differences between AAW and CAW patient samples in the expression of 84 genes previously implicated in breast cancer aggressiveness, estrogen receptor signaling, resistance to chemotherapy, and patient prognosis using the Human Breast Cancer and Estrogen Receptor Signaling RT^2^ Profiler PCR Array from SABiosciences (Table 2). Based on gene Delta C_T_ values, the Wilcoxon–Mann–Whitney test determined that 20 of the 84 genes examined were significantly differentially expressed (p ≤ 0.05) between the AAW and CAW patients, with a greater than 2-fold change in expression (Table 3; Figure 1). Of these 20 genes, only *CDKN2A* displayed increased expression in women of African-American descent.

**Table 2:** Human breast cancer and extrogen recptor RT2 profiler PCR array

**Description**	**Accession #:**
*Genes Associated with Breast Cancer Prognosis*	
Androgen receptor (AR)	NM_000044
Antigen identified by monoclonal antibody Ki-67 (MKI67)	NM_002417
B-cell CLL/lymphoma 2 (BCL2)	NM_000633
BCL2-associated agonist of cell death (BAD)	NM_004322
BCL2-associated athanogene (BAG1)	NM_004323
Cadherin 1, type 1, E-cadherin (CDH1)	NM_004360
Catenin (cadherin-associated protein), beta 1, (CTNNB1)	NM_001904
Cathepsin B (CTSB)	NM_001908
Clusterin (CLU)	NM_001831
Collagen, type VI, alpha 1 (COL6A1)	NM_001848
Cyclin A1 (CCNA1)	NM_003914
Cyclin A2 (CCNA2)	NM_001237
Cyclin D1 (CCND1)	NM_053056
Cyclin E1 (CCNE1)	NM_001238
Cyclin-dependent kinase inhibitor 1A (CDKN1A)	NM_000389
Cyclin-dependent kinase inhibitor 1B (CDKN1B)	NM_004064
Cyclin-dependent kinase inhibitor 2A (CDKN2A)	NM_000077
Epidermal growth factor receptor (EGFR)	NM_005228
Estrogen receptor 1 (ESR1)	NM_000125
Estrogen receptor 2 (ESR2)	NM_001437
Fas ligand (TNF superfamily, member 6) (FASLG)	NM_000639
FOS-like antigen 1 (FOSL1)	NM_005438
GATA binding protein 3 (GATA3)	NM_002051
Gelsolin (GSN)	NM_000177
Inhibitor of DNA binding 2 (ID2)	NM_002166
Insulin-like growth factor binding protein 2 (IGFBP2)	NM_000597
Integrin, alpha 6 (ITGA6)	NM_000210
Integrin, beta 4 (ITGB4)	NM_000213
Interleukin 2 receptor, alpha (IL2RA)	NM_000417
Interleukin 6 (IL6)	NM_000600
Interleukin 6 receptor (IL6R)	NM_000565
Interleukin 6 signal transducer (IL6ST)	NM_002184
Jun oncogene (JUN)	NM_002228
Kallikrein-related peptidase 5 (KLK5)	NM_012427
Keratin 19 (KRT19)	NM_002276
Kruppel-like factor 5 (KLF5)	NM_001730
Mitogen-activated protein kinase kinase 7 (MAP2K7)	NM_145185
Mucin 1, cell surface associated (MUC1)	NM_001018016
Nerve growth factor (NGF)	NM_002506
Nerve growth factor receptor (NGFR)	NM_002507
Prostaglandin-endoperoxide synthase 2 (PTGS2)	NM_000963
TNF receptor superfamily, member 6 (FAS)	NM_000043
V-erb-b2 erythroblastic leukemia viral oncogene homolog 2 (ERBB2)	NM_004448
*Genes Associated with Estrogen Receptor Signaling*	
Non-metastatic cells 1 (NME1)	NM_000269
Phosphatase and tensin homolog (PTEN)	NM_000314
Plasminogen activator, urokinase (PLAU)	NM_002658
Progesterone receptor (PGR)	NM_000926
Serpin peptidase inhibitor, clade B, member 5 (SERPINB5)	NM_002639
Serpin peptidase inhibitor, clade E, member 1 (SERPINE1)	NM_000602
Thrombospondin 1 (THBS1)	NM_003246
Topoisomerase (DNA) II alpha (TOP2A)	NM_001067
Transforming growth factor, alpha (TGFA)	NM_003236
Tumor protein p53 (TP53)	NM_000546
Tyrosine kinase with immunoglobulin-like and EGF-like domains 1 (TIE1)	NM_005424
Vascular endothelial growth factor A (VEGFA)	NM_003376
*Genes Associated with Response to Chemotherapy*	
Cathepsin D (CTSD)	NM_001909
Complement component 3 (C3)	NM_000064
Heat shock 27 kDa protein 1 (HSPB1)	NM_001540
Keratin 18 (KRT18)	NM_000224
Serpin peptidase inhibitor, member 3 (SERPINA3)	NM_001085
Solute carrier family 7, member 5 (SLC7A5)	NM_003486
Stanniocalcin 2 (STC2)	NM_003714
Trefoil factor 1 (TFF1)	NM_003225
*Genes Associated with Breast Cancer Diagnosis and Progression*	
BCL2-like 2 (BCL2L2)	NM_004050
CD44 molecule (CD44)	NM_000610
Claudin 7 (CLDN7)	NM_001307
Cytochrome P450, family 19, subfamily A, polypeptide (CYP19A1)	NM_000103
Deleted in liver cancer 1 (DLC1)	NM_006094
Fibroblast growth factor 1 (FGF1)	NM_000800
Fibronectin leucine rich transmembrane protein 1 (FLRT1)	NM_013280
Gamma-aminobutyric acid (GABA) A receptor, pi (GABRP)	NM_014211
GNAS complex locus (GNAS)	NM_080425
High-mobility group box 1 (HMGB1)	NM_002128
Metallothionein 3 (MT3)	NM_005954
Nuclear transcription factor Y, beta (NFYB)	NM_006166
Pregnancy-associated plasma protein A, pappalysin 1 (PAPPA)	NM_002581
Ras-related C3 botulinum toxin substrate 2 (RAC2)	NM_002872
Ribosomal protein L27 (RPL27)	NM_000988
Secretoglobin, family 1D, member 2 (SCGB1D2)	NM_006551
Secretoglobin, family 2A, member 1 (SCGB2A1)	NM_002407
Small proline-rich protein 1B (SPRR1B)	NM_003125
Thrombospondin 2 (THBS2)	NM_003247
Tumor necrosis factor, alpha-induced protein 2 (TNFAIP2)	NM_006291
V-kit Hardy-Zuckerman 4 feline sarcoma viral oncogene homolog (KIT)	NM_000222
*Housekeeping Genes*	
Actin, beta (ACTB)	NM_001101
Beta-2-microglobulin (B2M)	NM_004048
Glyceraldehyde-3-phosphate dehydrogenase (GAPDH)	NM_002046
Hypoxanthine phosphoribosyltransferase 1 (HPRT1)	NM_000194
Ribosomal protein L13a (RPL13A)	NM_012423

**Table 3:** Breast cancer genes differentially expressed between CAW and AAW patient populations

**Gene**	**Description**	**CAW**^a^	**AAW**^b^	**CAW/AAW**^c^	**P-valued**
** *Genes Associated with Breast Cancer Prognosis* **					
AR	Androgen receptor	0.99	0.38	2.60	0.002
BCL2	B-cell CLL/lymphoma 2	0.65	0.20	3.28	0.010
CCND1	Cyclin D1	0.78	0.28	2.75	0.030
CDKN1A	Cyclin-dependent kinase inhibitor 1A	2.07	0.90	2.29	0.002
CDKN1B	Cyclin-dependent kinase inhibitor 1B	1.68	0.74	2.26	0.001
CDKN2A	Cyclin-dependent kinase inhibitor 2A	1.35	3.55	0.38	0.046
ERBB2	V-erb-b2 erythroblastic leukemia viral oncogene homolog 2	1.61	0.63	2.56	0.012
ESR1	Estrogen receptor 1	1.79	0.46	3.91	0.023
GATA3	GATA binding protein 3	1.57	0.17	9.33	0.001
IGFBP2	Insulin-like growth factor binding protein 2, 36 kDa	1.53	0.73	2.11	0.005
IL6ST	Interleukin 6 signal transducer	1.15	0.44	2.64	0.005
KRT19	Keratin 19	1.44	0.71	2.03	0.012
MUC1	Mucin 1, cell surface associated	1.77	0.36	4.94	0.004
PGR	Progesterone receptor	0.77	0.33	2.34	0.012
SERPINE1	Serpin peptidase inhibitor, clade E, member 1	7.59	3.18	2.38	0.014
** *Genes Associated with Response to Chemotherapy* **					
HSPB1	Heat shock 27 kDa protein 1	0.98	0.46	2.14	0.003
SERPINA3	Serpin peptidase inhibitor, clade A, member 3	0.43	0.20	2.16	0.017
STC2	Stanniocalcin 2	0.79	0.38	2.05	0.019
** *Genes Associated with Breast Cancer Progression* **					
CLDN7	Claudin 7	1.76	0.66	2.67	0.006
DLC1	Deleted in liver cancer 1	4.27	0.94	4.52	0.023

^a^ Average Gene Expression of Caucasion-American Women (CAW, n = 12).

^b^ Average Gene Expression of African-Amercian Women (AAW, n = 12).

^c^ Gene Expression Fold Change Relative to the Average Gene Expression of AAW.

^d^ P-values calculated using the Wilcoxin Rank Sum Test.

**Figure 2:**
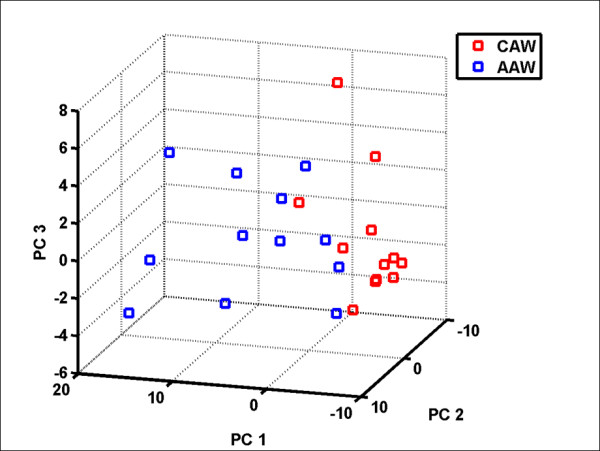
Semi-Supervised Principal Component Analysis significantly (p < 0.005) segregated the AAW and CAW breast cancer patients into two distinct groups based on combinations of the first three principal components (PCs).

We then visualized the ability of the 20 differentially expressed genes to segregate the AAW and CAW patients into distinct molecular subgroups. SSPCA was performed by applying only the 20 genes identified by the Wilcoxon-Mann–Whitney test in principal component analysis. Application of the Kolmogorov-Smirnov normality test to the first three identified PCs determined that all three principle components (PCs) had an approximate normal distribution (p < 0.05) across both patient ethnicities. Based on the multivariate Hotelling T-Squared test, as would be expected the 20-gene combination significantly separated the breast cancer patients into two distinct subgroups (Figure 2) using PC1 (p < 0.001), PC1 and PC2 (p < 0.001), or the first three PCs (p < 0.001).

A comprehensive review of the literature determined that of the 20 differentially expressed genes, 70% (*AR, BCL2, CCND1, ESR1, GATA3, IGFBP2, IL6ST, KRT19, MUC1, PGR, SERPINE1, HSPB1, SERPINA3, and STC2*) have been previously associated with estrogen signaling and/or estrogen receptor 1 expression. To determine if similar associations with estrogen signaling could be detected in this study, Pearson’s correlation was used to determine the strength and direction of any underlying relationships with *ESR1* expression. Of the above listed genes, *ESR1* was positively associated with the expression of *BCL2, GATA3, IL6ST, MUC1, SERPINE1, AR*, and *HSPB1* expressions in women of African-American descent, with BCL2 displaying the strongest association (Table 3). In contrast, only the expression of *KRT19* and CCND1 were correlated with *ESR1* expression in the Caucasian population (Table 4).

**Table 4:** Genes previously associated with estrogen signalling

	**CAW**		**AAW**	
**Gene**	**r**	**P - Value**	**r**	**P - Value**
BCL2	NS	NS	0.95	<0.001
CCND1	0.65	0.0228	NS	NS
GATA3	NS	NS	0.76	0.0043
IGFBP2	NS	NS	NS	NS
IL6ST	NS	NS	0.85	0.0043
KRT19	0.76	0.0041	NS	NS
MUC1	NS	NS	0.84	0.0006
SERPINE1	NS	NS	0.86	0.004
AR	NS	NS	0.80	0.0017
HSPB1	NS	NS	0.82	0.001
SERPINA3	NS	NS	NS	NS
STC2	NS	NS	NS	NS

*NS* Not Significant.

In addition to estrogen signaling genes, 4 of the 20 identified genes have been implicated in resistance to targeted therapies (*ERBB2, ESR1, PGR,* and *AR*). Previous studies have reported that tumor specimens from women of African-American heritage are significantly more likely to lack expression of the hormone receptors *ESR1, PGR,* and *AR*, compared to other ethnicities. In the current study we found significantly lower expression of these receptors (6.7-fold) in the AAW compared to CAW patients (Table 3) for *PGR* (58%; 17%), and *ESR1* (58%; 17%) respectively. In addition, a larger percentage of AAW than CAW patients had no measurable expression of the *PGR* (33%; 8%) and *ESR1* (50%; 8%) genes respectively. AAW patients were also less likely to express *ERBB2* (42%; 8%).

In addition to estrogen signaling and resistance to therapy, 7 of the identified genes have been previously associated with cell cycle regulation (*CCND1, CDKN1A, CDKN1B,* and *CDKN2A*) and breast cancer aggressiveness (*CLDN7* and *DLC1*) (Table 3).

## Discussion

Women of African-American descent are diagnosed with breast cancer at a younger age and clinical stage than their Caucasian counterparts. In addition, although CAW have a higher incidence of breast cancer, AAW have poorer survival rates. While multiple studies have shown that these disparities in health outcomes are, in part, due to such societal factors as social economic status, access to appropriate health care, diet and religious beliefs, population-based studies showed that differences in patient diagnosis and survival remained even after taking such factors into account, suggesting biological underpinnings in race may be responsible. Identification of genetic contributors that may be driving the racial differences in clinical outcome is critical as such factors may alter preventative medicine, cancer screening practices, and therapeutic guidelines. The aim of the current study was to gain a more in-depth understanding of gene expression differences between AAW and CAW breast cancer patients that may contribute to the poorer outcome of AAW patients.

Interestingly, even though patients were matched on both age and stage at diagnosis, tumor tissues from women of African decent were significantly more likely to be of higher grade. Although AAW tumors were also more likely to display the triple negative (ER-, PR-, HER2-) phenotype, these observations did not reach statistical significance. Higher grade and a triple negative phenotype are known negative predictors of breast cancer prognosis. Thus, these findings are in agreement with past studies and support that AAW have a molecular predisposition for a more aggressive breast cancer phenotype.

In the current study the expression of 84 genes (Table 2) previously implicated in breast cancer aggressiveness, estrogen receptor signaling, resistance to chemotherapy, and patient prognosis were examined in formalin-fixed, paraffin-embedded (FFPE) tissues obtained from age- and stage-matched AAW and CAW patients. Gene expression analysis of archival tissues has traditionally been problematic due to nucleotide degradation resulting from tissue processing. However, immense progress has been made in both the RNA isolation from and expression analysis of FFPE tissues [36,37]. In fact, several studies specifically comparing gene expression profiles from matched snap-flash frozen and FFPE tissues demonstrated significant concordance (r = 0.92, P < 0.0001) [29,38], opening the use of archival tissues for gene expression analysis.

The current study identified 20 genes that had a significant and greater than 2-fold change in expression between AAW and CAW patients using the Wilcoxon-Mann–Whitney test (Table 3). As illustrated in Figure 1, virtually all of the genes identified displayed increased expression in Caucasian compared to African-American women. Only CDKN1A displayed a significantly higher expression in AAW. To determine if the differential expression of these genes could discriminate between African-American and Caucasian patients, we performed SSPCA. SSPCA is advantageous over general principal component analysis in that, by only using those genes associated with ethnic background, patient clustering can be visualized without background noise resulting from genes that are not differentially expressed. This analysis determined that the AAW and CAW breast cancer patients could be visually clustered based only on the expression of these 20 genes using combinations of principal component PC1, PC2, and PC3 (Figure 2, p < 0.001 for all PC combinations).

In support of previous studies examining molecular differences between African-American and Caucasian-American women, our study suggests that AAW have a gene expression-based predisposition for a more aggressive and treatment resistant tumor phenotype than CAW. The 20 differentially expressed genes identified (Table 3) have been implicated in cell cycle regulation, response to therapy, estrogen signaling and breast cancer aggressiveness. Abnormalities in the levels of G_1_/S phase cell-cycle regulatory proteins have been previously associated with breast cancer prognosis and response to therapy [39-44]. In our study the expression of *CCND1, CDKN1A* and *CDKN1B* was significantly elevated, while *CDKN2A* was significantly lower, in Caucasian-American compared to African-American patients. *CCND1* (Cyclin D) drives the G_1_/S phase transition through binding with cyclin dependent kinase 4 (CDK4) and cyclin dependent kinase 6 (CDK6), which then phosphorylates retinoblastoma (pRb), inducing downstream *Cyclin E* transcription [45]. In contrast CDKN1A (p21), CDKN1B (p27), and CDKN2A (p16) are cyclin dependent kinase inhibitor proteins (CDK inhibitors) involved in cell cycle arrest through inhibition of CDK4, CDK6, and cyclin dependent kinase 2 (CDK2) [45]. As a whole, the lower expression of *CCND1, CDKN1A* and *CDKN1B*, and higher expression of *CDKN2A* in AAW versus CAW patients would hypothetically result in decreased cellular proliferation in AAW tumor specimens, yet AAW patient tissues had a statistically significant higher grade than CAW specimens in this study. Interestingly, other studies have also observed this same contradiction [20], and in fact, have noted a distinct inverse relationship between *cyclin D1*[20,46,47] and *p16*[20,48,49] levels with poorer clinical outcome, a more aggressive cancer phenotype, and resistance to multiple chemotherapeutic agents. The collective findings of our and these studies suggest that deregulation of cell-cycle G_1_ regulatory genes is common in women of African-American heritage and may contribute to the poorer outcome of this ethnic group.

In addition to differences in cell-cycle regulatory genes, we also noted distinct differences in the expression of genes previously implicated in treatment response between AAW and CAW patient tumor specimens. As clearly illustrated in Figure 2, AAW patients had significantly lower (P < 0.05) expression of *ESR1* (ER), *PGR* (PR), and *ERBB2* (HER-2), compared to CAW patients with a greater percentage of AAW patients exhibiting no detectable expression of *ESR1* (8% vs. 50%) or *PGR* (8% vs. 33%). A similar trend was observed in the tumoral protein receptor status for ER, PR, and HER2 (Table 1). These data add to the growing body of evidence that women of African-American descent are statistically more likely to be estrogen and progesterone receptor-negative [17,50-53]. ER + and PR + tumor status is typically associated with increased survival and enhanced response to hormonal therapy. In contrast, lack of estrogen and progesterone receptor expression has been associated with a more aggressive phenotype and worse clinical outcome, as lack of estrogen or progesterone receptor status precludes treatment with tamoxifen or trastuzumab [50,51]. Unlike the racial differences observed for *ESR1* and *PGR*, no such association has been described for *ERBB2*. In two separate multiethnic population-based studies conducted by Elledge et al. [52] and Porter et al. [20], HER-2 levels were found to be similar between all ethnic groups examined. However, the protein expression of HER-2 was assessed through immunohistochemistry in both of these studies, and thus differences in findings may reflect a dissociation between protein and mRNA levels of HER-2/ERRB2. While elevated *ERRB2* expression has been associated with increased disease recurrence, metastasis, and shorter survival, enhanced survival is also observed for these patients when treated with HER-2 targeted therapies such as Herceptin [54,55] and Tykerb [54,56]. These results suggest AAW may harbor gene expression profile differences that increase tumor resistance to current targeted hormone and HER2 therapies. Collectively, this data supports that there are inherent gene expression differences in *ESR1PGR*, and *ERBB2* between women of African and Caucasian-American decent that potentially contributes to the triple negative phenotype (ER-, PR-, and HER2-) and poorer outcome often observed for AAW.

Interestingly, 70% of the genes differentially expressed between African-American and Caucasian-American women in our study have been implicated in estrogen signaling, including *AR, BCL2, CCND1, ESR1, GATA3, IGFBP2, IL6ST, KRT19, MUC1, PGR, SERPINE1, HSPB1, SERPINA3,* and *STC2*, all of which displayed decreased expression in AAW compared to CAW patients (Table 4). The expression of *AR*[57,58], *BCL2*[57-59], *CCND1*[57,58,60], *GATA3*[57,58,61,62], *IL6ST*[57,58], *MUC1*[58,63,64], *PGR*[57,65], *SERPINE1*[66,67], *HSPB1*[58,68,69] and *STC2*[57,58,69,70] have been positively associated with *ESR1* expression and/or upregulated by *ESR1*. Other studies have found estrogen can directly upregulate transcription of *AR*[57], *GATA3*[57], *IGFBP2*[71,72], *KRT19*[73], and *MUC1*[64], *PGR*[57,74,75], *SERPINE1*[76], *HSPB1*[77-79], *SERPINA3*[80,81], *SERPINE1*[76], and *STC2*[57,69,70]. In support of these studies, we also found that the expression of *AR, BCL2, GATA3, IL6ST, MUC1, PGR, SERPINE1*, and *HSPB1*, were significantly associated with *ESR1* expression in AAW; although, only *KRT19* and *CCND1* were positively correlated with *ESR1* levels in American women of Caucasian descent. Interestingly, lower levels of *BCL2*[59,82,83], *CCND1*[46,84], *GATA3*[62,85,86], *IL6ST*[87], *KRT19*[88], *MUC1*[63,64,89], *PGR*[65,90], *SERPINE1*[66], *SERPINA3*[69], *STC2*[91], while higher levels of *AR*[65,92], *SERPINA3*[69], and *STC2*[69] have been associated with enhanced response to hormone therapy in sex steroid positive tumors. These results suggest that women of African-American ethnicity are more prone to displaying negative or low expression of *ESR1* and its associated estrogen response genes, which have been correlated with resistance to hormone therapy and worse clinical outcome.

In addition to genes involved in cell cycle, treatment response, and estrogen signaling, we also determined that the cell adhesion-related genes *CLDN7* and *DLC1* were significantly decreased in the AAW patients (Table 3, Figure 1). *CLDN7* is a member of the claudin family of transmembrane proteins, which are critical structural and molecular components of tight junctions [93,94], necessary for cell-cell adhesion. Studies suggest that loss of tight junctions from down-regulation of claudins in various cancers, results in loss of cohesion, increased invasiveness, and cell dedifferentiation [95]. In support of these findings, loss or decreased expression of *CLDN7*, which is expressed constitutively during mammary epithelium development [96], has been significantly associated with higher histological grade, loss of cellular cohesion, and increased metastasis in breast carcinoma [97,98]. In light of this data, *CLDN7* has been proposed as a breast cancer tumor-suppressor gene. Like *CLDN7, DLC1* is has been considered a tumor suppressor gene involved in the regulation of the actin cytoskeleton, cell polarity, inter-cell focal adhesion, cell migration, and apoptosis [99-101] through negative regulation of Rho signaling pathways [99]. *DLC1* is expressed in multiple tissues including the brain, heart, kidney, liver, lung, skin, spleen, and testis [102]. Studies have found that the mRNA levels of *DLC1* are diminished in various cancers [103], including breast, through loss of heterozygosity or heterozygous gene deletions [104]. Furthermore, several studies investigating the role of *DLC1* in breast cancer found that forced expression of *DLC-1* in *DLC-1* negative breast cancer cell lines resulted in growth inhibition, reduction in colony formation, and abolishment of *in vivo* tumorigenicity [103,105], whereas downregulation of *DLC1* expression enhanced cell motility and chemotactic behavior [106]. These studies suggest that loss or reduced expression of *CLDN7* and *DLC1,* as was observed in tumor specimens from AAW, may lead to increased cell motility, migration, metastasis and dedifferentiation, all of which may contribute to the worse clinical prognosis observed for AAW.

The large number of gene expression differences observed in this study between AAW and CAW patients supports that women of African-American decent may harbor differences in gene expression profiles that predispose them to increased tumor grade, a triple negative (ER-, PR-, HER2-) phenotype, and worse clinical disease outcome. Future studies are needed to determine if these gene expression profiles are grade and receptor status specific or represent other attributing factors to AAW poorer prognosis.

## Conclusions

The main objective of this study was to identify gene expression differences between AAW and CAW that may contribute to the poor clinical outcome observed for women of African-American descent. While the small sample size examined in this study is a limiting factor, the use of only age- and stage-matched tumor specimens strengthens findings from this study. This study demonstrated that tumor specimens from AAW were significantly more likely to display aberrations in G_1_/S cell-cycle regulatory genes, lack or exhibit low expression of *ESR1, PGR,* and *ERBB2* with a decrease in estrogen signaling pathway targets, and display a decrease in the expression of cell-adhesion genes. These factors have been collectively linked with a more aggressive cancer phenotype, resistance to multiple chemotherapeutic agents, enhanced metastatic potential, and poorer clinical outcome, further supporting the hypothesis that women of African-American ancestry have ethnic differences in gene expression patterns that predisposes them to a highly aggressive and treatment-resistant breast cancer phenotype.

## Authors’ contributions

JMG carried out the data interpretation, participated in the statistical analysis, and drafted the manuscript. ADS processed the tissue samples, carried out the gene expression analysis, and participated in drafting the manuscript. QH carried out the statistical analysis and participated in drafting the manuscript. MRS participated in sample acquisition. SBP participated in the study design and carried out the patient data acquisition. MRJ and WEG jointly conceived and coordinated the study. All authors reviewed and approved of the final manuscript.

## Competing interests

The authors declare that they have no competing interests.
